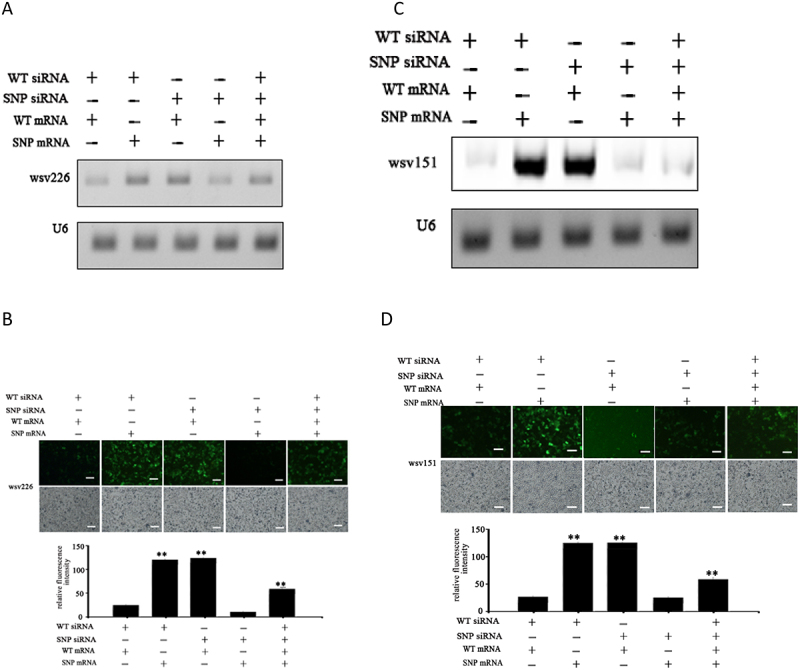# Correction

**DOI:** 10.1080/15476286.2024.2437203

**Published:** 2024-12-12

Article title: Synonymous SNPs of viral genes facilitate virus to escape host antiviral RNAi immunity

Authors: Y. Sun., Y. Zhang., and X. Zhang

Journal: ***RNA Biology***

Bibliometrics: Volume 16, Number 12, pages 1697 - 1710

DOI: https://doi.org/10.1080/15476286.2019.1656026

This article was previously published with an error in “Figure 3 (C and D)” that was overlooked. This has now been corrected and republished in the original article.

**Figure 3:**